# Seizing the policy moment in crop biotech regulation: an interdisciplinary response to the Executive Order on biotechnology

**DOI:** 10.3389/fbioe.2023.1241537

**Published:** 2023-08-07

**Authors:** Jill Furgurson, Nick Loschin, Eric Butoto, Modesta Abugu, Christopher J. Gillespie, Rebekah Brown, Greg Ferraro, Nolan Speicher, Ruthie Stokes, Asa Budnick, Katrina Geist, Rex Alirigia, Amaja Andrews, Amanda Mainello

**Affiliations:** ^1^ North Carolina State University Forestry and Environmental Resources, Raleigh, NC, United States; ^2^ North Carolina State University Genetic Engineering and Society Center, Raleigh, NC, United States; ^3^ North Carolina State University Applied Ecology, Raleigh, NC, United States; ^4^ North Carolina State University Crop and Soil Sciences, Raleigh, NC, United States; ^5^ North Carolina State University Horticultural Science, Raleigh, NC, United States; ^6^ North Carolina State University Entomology and Plant Pathology, Raleigh, NC, United States; ^7^ North Carolina State University Food, Bioprocessing and Nutrition Sciences, Raleigh, NC, United States; ^8^ North Carolina State University Agricultural and Resource Economics, Raleigh, NC, United States; ^9^ North Carolina State University Communication, Rhetoric, and Digital Media, Raleigh, NC, United States; ^10^ North Carolina State University Biochemistry, Raleigh, NC, United States; ^11^ North Carolina State University Plant Biology, Raleigh, NC, United States; ^12^ North Carolina State University Anthropology, Raleigh, NC, United States

**Keywords:** genetic engineering, coordinated framework, GMO, public engagement, policy

## 1 Introduction

The release of the Biden Administration’s Executive Order on Biotechnology and Biomanufacturing signals that a policy window is open for significantly revising and evolving the existing regulatory framework for agricultural biotechnology products. In the fall of 2022, the Executive Order called for the identification of areas of ambiguity, gaps, or uncertainties in the regulation of biotechnology (Executive Order 14801, 2022). Despite the fact that genetically engineered crops and products can yield scientific controversy and public contestation (Lefebvre et al., 2019; Gordon et al., 2021; Cummings and Peters, 2022), the Executive Order gives little explicit direction for their development. Appropriate governmental oversight of these products is necessary to assess potential benefits and risks, facilitate international trade, and build public trust (NASEM, 2016).

The governance of US crop biotechnology has been in policy flux since genetic engineering was first put forth as a means of addressing challenges in agriculture and food security and is fraught with complexity and controversy. Oversight in the form of policies and frameworks for regulation should assist society in assessing the potential benefits, risks, and other concerns arising from new technologies (Carroll et al., 2016). And public engagement in the processes of development of agricultural biotechnology has the potential to strengthen the credibility of both developers and regulators, while providing a means to address issues related to public trust and socio-cultural concerns (Kjeldaas et al., 2023). However, current regulation lacks the transparency and public deliberation needed to incorporate the views of wider society. The committee of the 2017 NASEM report, Preparing for Future Products of Biotechnology, describes the gaps and redundancies, as well as the complexity of the current system: “This complexity can cause uncertainty and a lack of predictability for developers of future biotechnology products and creates the potential for loss of public confidence in oversight of future biotechnology products” (NASEM, 2017, p. 6).

Many policy actors are vying to shape new regulatory frameworks, with distinct narratives emerging from biotechnology developers and other experts in regard to governance (Benbrook, 2016; Kuzma, 2022; Jenkins et al., 2023), the emphasis on product vs. process (Carroll et al., 2016; Marchant and Stevens, 2016; Gould et al., 2022), and the competing values of diverse publics (Jordan et al., 2017; Selfa et al., 2021; Strobbe et al., 2023). As a group of interdisciplinary scholars examining the impacts of biotechnology on our food, energy, and water systems through the Genetic Engineering and Society Center at North Carolina State University, we are uniquely positioned to focus a response to the Executive Order on the governance of gene edited foods and crops. Following a brief review of the regulatory policies and frameworks leading to the present policy moment, we will discuss how systematic data sharing and legitimate public engagement present opportunities for greater transparency and trust building in the regulation of agricultural biotechnology.

## 2 Historical framework

Throughout the history of genetic engineering, the US regulatory system has struggled to adapt to emerging technological advances. Federal regulatory policy is encapsulated by The Coordinated Framework for the Regulation of Biotechnology (CFRB), which was established in 1986. The CFRB assigns responsibility for governance to three US agencies, the United States Department of Agriculture (USDA), Food and Drug Administration (FDA), and Environmental Protection Agency (EPA), whose authority is defined by existing law. The CFRB has since gone through multiple revisions, and these agencies have adapted their regulatory guidelines in response to new scientific techniques and input from key actors.

The most recent attempt to “modernize” the regulatory system, the USDA Sustainable, Ecological, Consistent, Uniform, Responsible, Efficient (SECURE) rule, is neither responsible nor consistent. The rule was created in 2020 following the development of novel gene-editing techniques such as CRISPR in order to streamline the regulation and approval of new GE products. The rule allows certain gene edited products, such as those achieved through single-base pair substitutions, to completely circumnavigate regulation and enter the market without any health and safety assessment. Gene editing techniques can result in mutations that are equivalent to those arrived at through mutagenesis breeding, but depending on the exact processes used they may also result in unintended alterations to the genome. It is technologically feasible for developers to sequence the entire genome of products to confirm that only the intended modifications are present. However, developers are not required to do so and if they were to discover unintended modifications it may require that the product be regulated (USDA-APHIS, 2020). The voluntary nature of the SECURE rule process leaves identifying the extent of genome modifications to the discretion of the developer (Latham et al., 2006; Eckerstorfer et al., 2019; Biswas et al., 2020). This emphasis on regulating the process of modification versus the final product means developers are not required to disclose information on these products and their modifications (Gordon et al., 2021; Hoffman, 2021). Critiques of the rule have centered around the loosening of restrictions and the increase in exemptions which allow for future genetically engineered crops and products to enter the market without regulation and formal risk assessments (Grossman, 2020; Kuzma and Grieger, 2020; Clark, 2021).

## 3 Opportunities for transparency and trust building in regulation

The public’s perception of genetically modified products is shaped by trust, and developers in turn recognize the importance of bolstering support for biotechnology by building public confidence (Kuzma, 2018; Diamond et al., 2020; Cummings and Peters, 2022). Writing about gene-edited foods (GEFs), Cummings and Peters (2022) note “that individuals’ willingness to eat, and purposeful avoidance of GEFs are primarily driven by their existing social values about food, science and technology, institutional trust, and awareness of GE foods. These antecedent, and more deeply seated core values supersede more immediate and topical concerns and opinions on the safety, cost, taste, and appearance of GEF products” (Cummings and Peters, 2022).

Currently, the public is behind a curtain of uncertainty when it comes to the approval of these novel products. The lack of transparency throughout the development and marketing process inhibits consumer autonomy and limits the ability of the public to participate in decision-making (Kuzma, 2022). We see two opportunities for building greater visibility and fostering trust in the regulatory system during this unique policy moment: the creation of a shared database ecosystem and reforming public engagement practices.

### 3.1 Shared database ecosystem

Section 1 of Biden’s Executive Order on Biotechnology cites the objective to “foster a biological data ecosystem that advances biotechnology and biomanufacturing innovation, while adhering to principles of security, privacy, and responsible conduct of research.” We argue that an additional aspect of this database should be to serve the public in making informed decisions. Aggregating and sharing data across local and federal agencies, research institutions, and public sectors is key to creating visibility in the development and distribution of genetically engineered products.

Although the CFRB in theory represents a coordinated effort at decision-making and risk assessment of gene edited products, data is currently siloed within each of these three distinct agencies. Depending on the exact genetic modification and when a new product is submitted for approval, it may be subject to regulation by one, two, or all three agencies. Agencies often work independently to provide their input on various risk assessments, sharing the limited data on their respective websites. There is a lack of transparency around decision-making and the approval process. In the absence of a centralized data ecosystem, consumers and other participants are left assembling pieces of a puzzle in an attempt to create a cohesive picture of the product.

There is a need to develop a shared and publicly accessible regulatory database to enhance transparency and trust and create accountability for regulators and developers (Kuzma and Grieger, 2020). At a minimum this database should centralize the required information that is already reported separately by CFRB agencies. Additionally, this database should contain the information deemed important by consumers, as well as mechanisms for monitoring the safety and efficacy of these technologies. Such a unified source of continuously updated information will encourage transparency in the risk assessment process; enhance data coordination across agencies; and provide a foundation for the bidirectional communication needed between institutions and the public.

The Nanodatabase offers one example of a public-facing tool that provides continuously updated safety information to consumers of nanoproducts in Europe (Hansen et al., 2016). We envision that a similar centralized information center for products developed through genetic engineering will foster public trust and understanding as a result of increased transparency around the scientific and regulatory aspects of this domain. We acknowledge it is no small task to create a database of technical information that is widely accessible to a variety of publics, however, interdisciplinary and cross-sector collaborations with specialists in web design, user experience, and science/technical communication can help build off the foundations already established. However difficult, we argue that in its potential to facilitate cross-talk between the technical, political, and public spheres, a centralized database fits well into the “new architecture” of science-society relations that is necessitated by recent advances in genetic engineering (Burall, 2018).

### 3.2 Public engagement

Section 2 of Biden’s Executive Order calls for consultation with outside stakeholders “as appropriate and consistent with applicable law … to advance the policies described in section 1”. This nod towards broader public engagement to advance the development of biotechnology falls short of calling for the types of engagement necessary to incorporate a wider array of values and worldviews into policy changes. Ideally, public engagement seeks and facilitates “the sharing and exchange of knowledge, perspectives, and preferences between or among groups who often have differences in expertise, power, and values” (NASEM, 2016). By expanding the scope of goals and concerns to include those of the society impacted by the biotechnology being developed, public engagement has the potential to lead to more useful and ethical science, policy, and innovation.

There is currently an absence of systematic broader engagement in the US regulation of biotechnology. As outlined in the Update to the CFRB, the predominant manner that the USDA (APHIS) “engages the public” regarding the Federal regulation of novel genetically engineered products is via the solicitation of comments (US Office of Science and Technology Policy, 2017). Currently, the public comment periods occur downstream in the innovation process, following product development and shortly after the receipt of a petition for nonregulated status. The FDA process for approving biotechnology products is even less open to the public–the developer submits safety reports and other data to the FDA and the evaluation process is completed without any broader public input. And under the SECURE rule, companies and developers may opt out of regulation entirely, avoiding any public engagement requirements. Although information is available online through the various agencies, all of the associated websites are difficult to navigate, lack transparency, and invoke a top-down “deficit model” approach to communication that can be particularly problematic in contexts of science and technology (Nisbet and Scheufele, 2009; Ahteensu, 2012; Goodwin, 2018).

The public comment periods above are far from a two-directional exchange of viewpoints and concerns. Building greater transparency and trust will require US regulators to systematically consider and incorporate the diverse knowledge and perspectives of society. Authentic engagement opportunities should be designed to bridge the gaps between science and society through increased dialogue and mutual learning (Gemen et al., 2015). In order to support more robust and trusted decision making “these kinds of exchanges can and should take place throughout the life cycle of an innovation” (Barnhill-Dilling and Delborne, 2021). And finally, because many of the public’s questions about these emerging technologies are political, ethical, or societal (Wirz et al., 2020), engagement must extend beyond the provision of facts to meaningfully incorporate values (Scheufele et al., 2021).

## 4 Discussion

The Biden Administration’s Executive Order presents an opportunity for systemic change in the regulation of biotechnology. Historically, the US regulatory system has been rigid in the face of new technologies, fostering an opaque and uncoordinated approach to decision-making. Emerging technologies such as gene-editing have been proposed as potential solutions to the wicked problems facing global agriculture and society at large. At the same time, they expose some of the glaring gaps in the current governance system. The present policy moment creates room for policymakers to fill these gaps through increased transparency via a shared database ecosystem and enhanced public engagement. Aspects of the regulatory process, such as data-sharing, require a centralized and transparent ecosystem for various participants to garner an understanding of a new product on the market. Developers, regulators, and policy makers need to adopt a bidirectional approach to better incorporate different perspectives, concerns, and goals. Failure to take advantage of this policy moment could negatively impact biotechnology research and development, and further corrode public trust. However, the creation of a regulatory system that provides access to shared data and integrates the values and perspectives of the public can support more trusted research and development while being responsive to societal concerns.
